# Cross-scale detection and cross-crop generalization verification of tomato diseases in complex agricultural environments

**DOI:** 10.3389/fpls.2025.1644271

**Published:** 2025-10-27

**Authors:** Jinghuan Hu, Jinying Li, Heyang Wang

**Affiliations:** ^1^ College of Information and Technology, Jilin Agricultural University, Changchun, China; ^2^ College of Horticulture, Jilin Agricultural University, Changchun, China

**Keywords:** tomato leaf disease, precision agriculture, agricultural artificial intelligence, multi-scale detection, transfer learning

## Abstract

In order to overcome the key challenges associated with detecting tomato leaf disease in complex agricultural environments, such as leaf occlusion, variation in lesion size and light interference, this study presents a lightweight detection model called ToMASD. This model integrates multi-scale feature decoupling and an adaptive alignment mechanism. The model innovatively comprises a dual-branch adaptive alignment module (TAAM) that achieves cross-scale lesion semantic alignment via a dynamic feature pyramid, a local context-aware gated unit (Faster-GLUDet) that uses a spatial attention mechanism to suppress background noise interference, and a multi-scale decoupling detection head (MDH) that balances the detection accuracy of small and diffuse lesions. On a dataset containing six types of disease under various weather conditions, ToMASD achieves an average precision of 84.3%,.by a margin of 4.7% to 12.1% over thirteen mainstream models. The computational load is compressed to 7.1 GFLOPs. Through the introduction of a transfer learning paradigm, the pre-trained weights of the tomato disease detection model can be transferred to common bean and potato detection tasks. Through domain adaptation layers and adversarial feature decoupling strategies, the domain shift problem is overcome, achieving an average precision of 92.7% on the target crop test set. False detection rates in foggy and strong light conditions are controlled at 6.3% and 9.8%, respectively. This study achieves dual breakthroughs in terms of both high-precision detection in complex scenarios and the cross-crop generalization ability of lightweight models. It provides a new paradigm for universal agricultural disease monitoring systems that can be deployed at the edge.

## 1 Introduction

Global agricultural diseases cause over 220 billion US dollars in economic losses each year. Among these, the early detection of leaf diseases in crops is a key part of precision plant protection (Astani et al., 2022). As an important economic crop cultivated worldwide, the control of tomato diseases directly affects crop yield, quality, and agricultural economic benefits. Although deep learning-based detection methods have made significant progress in single-crop scenarios, models generally perform poorly in cross-crop scenarios due to domain specificity and the scarcity of labelled data. This limitation is particularly evident in developing countries with limited resources: small-scale farmers cannot afford the development costs of multi-crop-specific models, and agricultural expert systems lack the ability to generalise heterologous disease features. This results in delayed disease warnings and the waste of prevention and control resources. Therefore, innovating and applying leaf disease detection technology is important for ensuring food security, promoting sustainable agricultural development, and advancing smart agricultural systems.

In natural farmland environments, leaf disease detection often faces multiple challenges, such as leaves shading each other, disease patches having diverse shapes, light conditions fluctuating and diseases having weak early symptoms. Traditional image processing methods mostly rely on colour space segmentation combined with texture feature extraction for classification. For example, Javidan et al. used K-means clustering to segment diseased areas and applied morphological operations to optimise edge detection, achieving a 98.97% accuracy rate under controlled lighting conditions (Javidan et al., 2023). Similarly, Bhagat et al. used a network search-based SVM for classification and detection of plant leaf diseases (Bhagat et al., 2020), while Rodríguez et al. collected potato canopy images using a drone equipped with a multispectral sensor (Rodríguez et al., 2021). They combined vegetation indices and machine learning algorithms to achieve early detection and severity assessment of potato late blight. Furthermore, Saleem et al. designed a leaf segmentation process based on the ExG index and the region-growing method and combined the proportion of the diseased area to assess severity (Saleem et al., 2024). However, traditional methods face insufficient generalization ability in complex farmland scenarios due to their heavy reliance on manual feature design and experience-driven parameter tuning, and are difficult to adapt to the multi-scale disease representation requirements in dynamic field environments.

The advent of CNN has precipitated a paradigm shift in the realm of agricultural disease detection, with end-to-end architectures predicated on single-stage detectors becoming the prevailing paradigm. This is primarily attributable to the enhanced efficiency exhibited by these architectures. In the context of tomato leaf disease detection, researchers frequently employ a combination of deep learning models and conventional image processing techniques to enhance the precision of lesion localization. For instance, Barbedo proposed a threshold segmentation approach based on the HSV color space and morphological processing to extract lesion areas and verified the feasibility of combining traditional methods with CNN (Convolutional Neural Network) (Barbedo, 2018). Similarly, R. et al. embedded an attention mechanism in a pre-trained residual CNN, combined with multi-spectral data to enhance lesion feature expression, improving the discrimination of lesion features in complex environments (R. et al., 2020). Furthermore, Cong et al. developed a lightweight Mask R-CNN variant, optimizing lesion boundary localization through the integration of superpixel segmentation and edge detection algorithms (Cong et al., 2023). As proposed by Shin et al., a feature extraction and data augmentation strategy was proposed, combining a CNN with RGB images (Shin et al., 2021). This strategy achieved an average accuracy of 92.18% in the detection of strawberry leaf powdery mildew. Despite the efficacy of the aforementioned method in certain contexts, it remains confronted with numerous challenges in the context of natural farmlands. The distinguishing characteristics of disease spots are often obscured by leaf occlusion in complex backgrounds, leading to ambiguity in identification (Debnath et al., 2023)This study adopts EfficientNetV2B2 as the lightweight backbone network to achieve efficient and accurate disease identification. sing the DL approach, tomato leaf disease identification achieves nearly 100% accuracy on a test dataset. Additionally, the presence of similar diseases can result in confusion regarding texture, and the identification of early disease spots with low contrast can be challenging (Saleem et al., 2024). Furthermore, the method’s accuracy in distinguishing cross-diseases with similar symptoms is often limited.

Currently, the field of plant disease detection generally faces the bottleneck of model generalization caused by domain differences. Existing research is mostly limited to customized training for single-crop diseases and is difficult to effectively transfer to heterologous crops. The detection of tomato leaf diseases in real-world agricultural settings is hindered by several key challenges: leaf occlusion and overlap.Different diseases share visual characteristics, leading to misclassification. To address this issue, this study proposes a cross-crop transfer learning framework that breaks through the domain shift limitations of cross-species disease recognition by sharing low-level feature representations and domain adaptation optimization strategies. Specifically, a CNN backbone model is trained with a tomato leaf disease dataset, and the transfer learning framework freezes the shallow feature extraction layers to retain the common texture and morphological features of crops and adapt to the specific phenotypes of target crop diseases, combined with adversarial training to minimize the distribution differences between domains. This achievement provides a cross-crop transfer learning paradigm for building a universal plant disease intelligent monitoring system and promotes the large-scale application of precision plant protection technology.

The primary contributions of this paper are as follows:

1. In order to address the challenges posed by the attenuation of features in small target disease spots and the failure to detect early disease spots, a novel dual-branch adaptive alignment module has been designed. Through dynamic feature alignment and cross-scale feature interaction, it significantly improves the detection accuracy and robustness of tomato leaf diseases in complex agricultural environments.
2. The Faster-GLUDet feature enhancement unit was integrated, which employs partial convolution and local context-aware gating mechanisms. This enhancement to the model’s noise suppression capabilities is achieved while maintaining its lightweight nature.
3. The construction of a multi-scale decoupled detection head was undertaken. The model achieves balanced detection of cross-scale diseases and efficient distinction between small disease spots, spreading lesions, and mixed diseases through hierarchical feature fusion and Group Normalization optimization.

## 2 Materials and methods

### 2.1 Data processing

#### 2.1.1 Data source

The tomato leaf disease dataset used in this study was sourced from the “Tomato Leaf Diseases Detect” standardized dataset released by the Roboflow open platform. It contains six typical disease categories (bacterial spot, early blight, late blight, leaf mold, target spot, and black spot) and healthy leaf samples, covering the early, middle, and late stages of disease development. In total, it includes 3,469 high-resolution RGB images. The six tomato leaf diseases of interest in this study are highly prevalent in major tomato-growing regions worldwide, causing yield losses of 20% to 65% (Lu et al., 2018; Panno et al., 2021). **Table 1** provides a detailed breakdown of the final image distribution across all categories after augmentation and splitting. The common Bean Dataset was captured at the Guoxin Modern Agricultural Base in Changchun City, Jilin Province, and the public dataset Bean Disease Dataset. The Potato dataset is from the public Potato disease dataset which includes three categories: health, early disease and late disease (https://gitcode.com/open-source-toolkit/829ec). The inclusion of these diverse datasets from different crops is intended to rigorously validate the transferability of the features learned by our model from tomatoes to other commercially important crops.

**Table 1:** Data distribution.

Data distribution	Training set	Validation set	Test set	All
Bacterial Spot	2841	465	221	3527
Early Blight	4994	618	669	6281
Healthy	1621	227	271	2119
Late Blight	2908	408	202	3524
Leaf Mold	2871	274	361	3506
Target Spot	2296	281	244	2821
Black Spot	3710	335	143	4418
All	21241	2608	2111	25960

#### 2.1.2 Data enhancement

**Figure 1:**
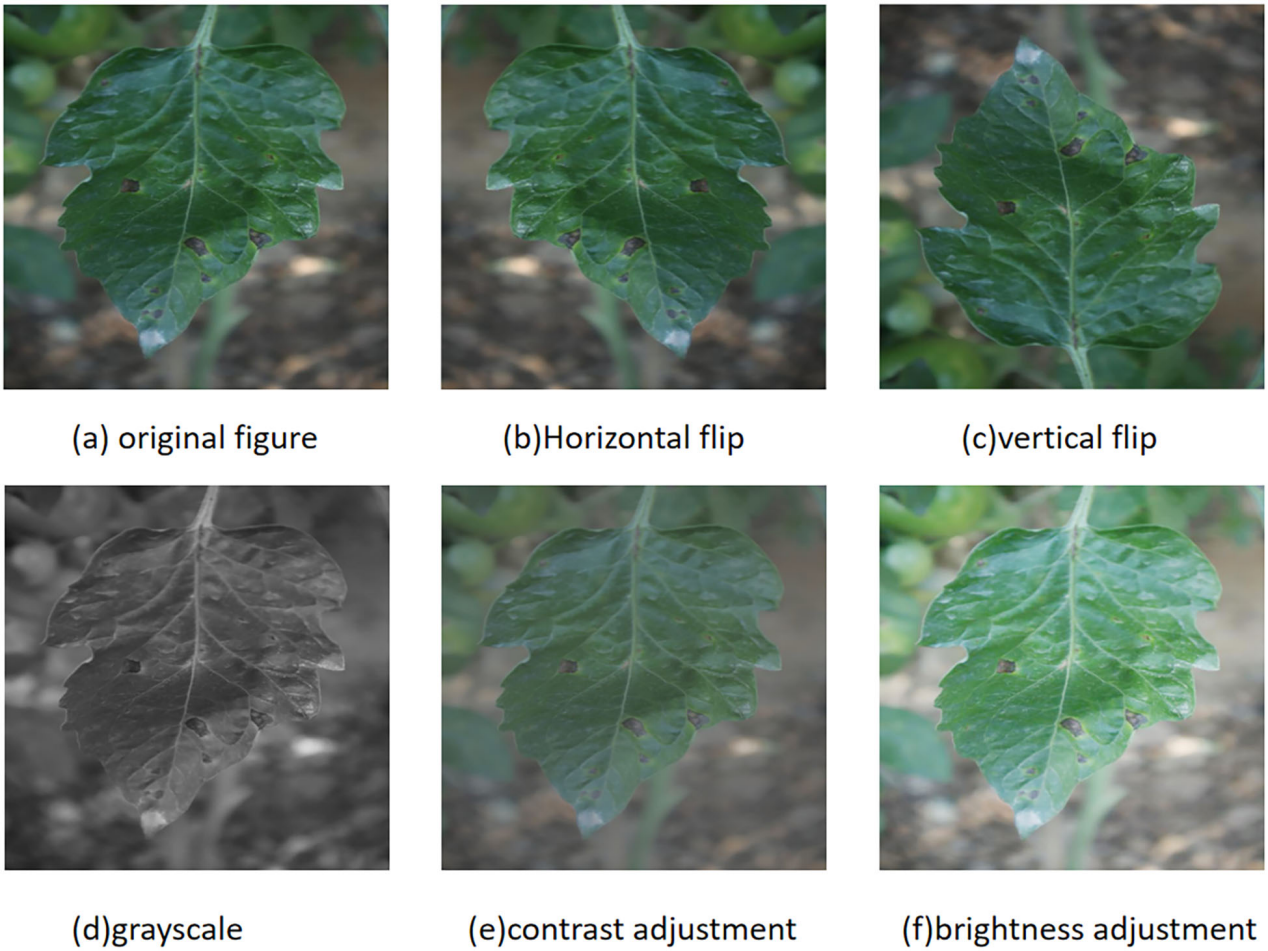
Data enhancement methods for tomato leaf images: **(a)** original image; **(b)** horizontal flip; **(c)** vertical flip; **(d)** grayscale conversion; **(e)** contrast adjustment; **(f)** brightness adjustment.

To enhance the robustness of the model, the dataset was expanded to 7370 images using data augmentation techniques. In order to prevent the original image and enhanced image from appearing simultaneously in the training set and validation set, the original image is initially divided into a training set, validation set, and test set in a ratio of approximately 8:1:1. Subsequently, five techniques including horizontal flipping, vertical flipping, grayscale, contrast adjustment, and brightness adjustment were randomly applied to the original data to enhance the image and labels. The enhanced example image is shown in **Figure 1**, and the label distribution is shown in **Table 1**.

In order to simulate the complex weather changes in the real tomato cultivation environment, this study adopts the RGB channel synthesis technology based on the atmospheric scattering model to generate enhanced images with controllable weather features on 50% of the typical samples in the training set. The synthesis formula is shown in Equation 1.


(1) $$\begin{array}{c} I(x)=J(x)\cdot t(x)+A\cdot (1-t(x)) \end{array}$$

**Figure 2:**
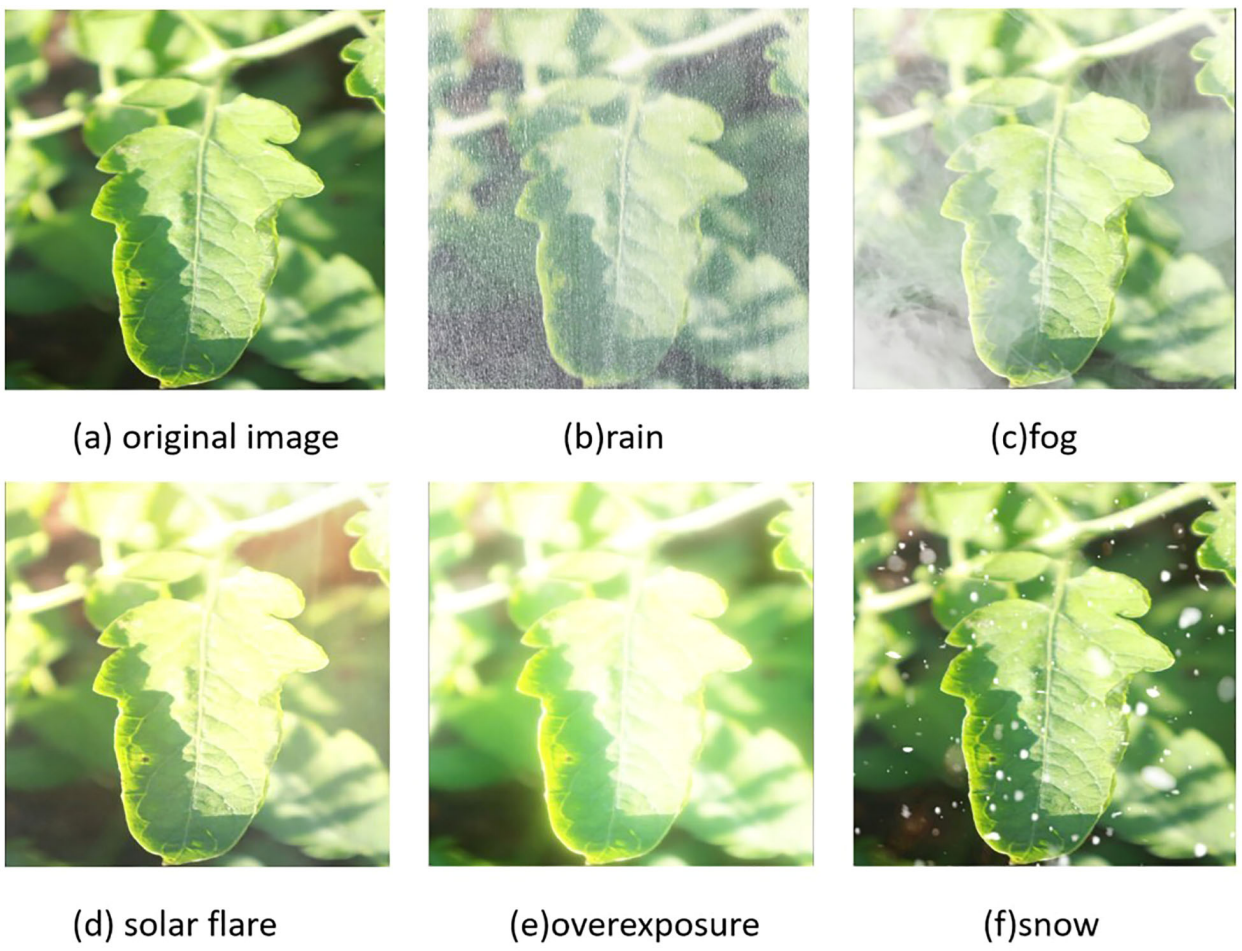
Randomly select a leaf image for weather synthesis: **(a)** original image; **(b)** rain simulation; **(c)** fog simulation; **(d)** solar flare simulation; **(e)** overexposure simulation; **(f)** snow simulation.

In this study, x denotes the pixel coordinate, I(x) signifies the synthesized image, J(x) represents the original image, the transmittance map t(x) is constrained within the interval [0.2, 0.8] and controls the weather intensity gradient, and the atmospheric light value A restricts the amplitude of illumination attenuation. **Figure 2** presents an image of medium-intensity synthetic weather.

### 2.2 Methodology

#### 2.2.1 Tomato multi-scenario adaptive scale detector

While general-purpose detectors like YOLOv11 have demonstrated strong performance on common datasets, their inherent architecture is not optimally designed for the unique challenges presented by complex agricultural environments, such as severe scale variation of lesions, leaf occlusions, and pervasive background noise. These limitations often lead to feature misalignment, reduced sensitivity to small objects, and compromised robustness under fluctuating lighting conditions.

To address these specific issues, we propose the Tomato Multi-scenario Adaptive Scale Detector (ToMASD), a novel lightweight architecture specifically engineered for high-precision disease detection in real-world field settings. The overarching design philosophy of ToMASD is to achieve an optimal balance between computational efficiency and detection accuracy by introducing three dedicated core modules that work in concert throughout the feature extraction and fusion pipeline.

**Figure 3:**
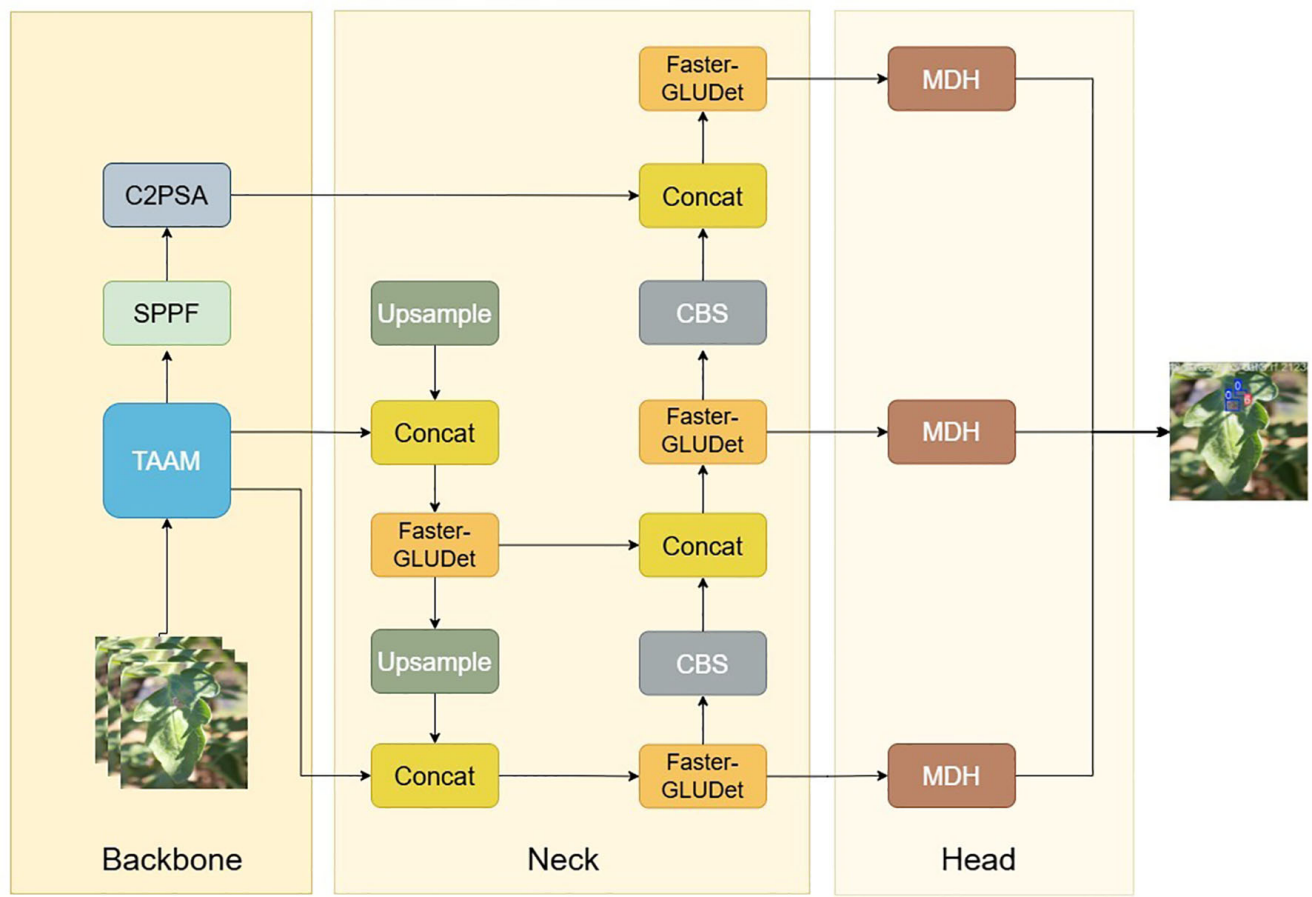
ToMASD model structure diagram.

As illustrated in **Figure 3**, the Two-branch Adaptive Alignment Module (TAAM) is integrated into the backbone network. Its purpose is to dynamically align and calibrate multi-scale features at the earliest stage, effectively mitigating the semantic misalignment between healthy and diseased tissue regions caused by occlusion and scale variance. The Faster-Gated Linear Unit (Faster-GLUDet) is embedded within the neck network. This module acts as an adaptive feature refiner, leveraging a gating mechanism to suppress irrelevant background noise. The Multi-scale Decoupling Head (MDH) is designed as the detection head. It replaces the conventional coupled head with a decoupled structure, allowing for independent optimization for classification and regression tasks at different feature scales. This synergistic design ensures that ToMASD is uniquely capable of handling the complexities of agricultural disease detection.

#### 2.2.2 Two-branch adaptive alignment module

**Figure 4:**
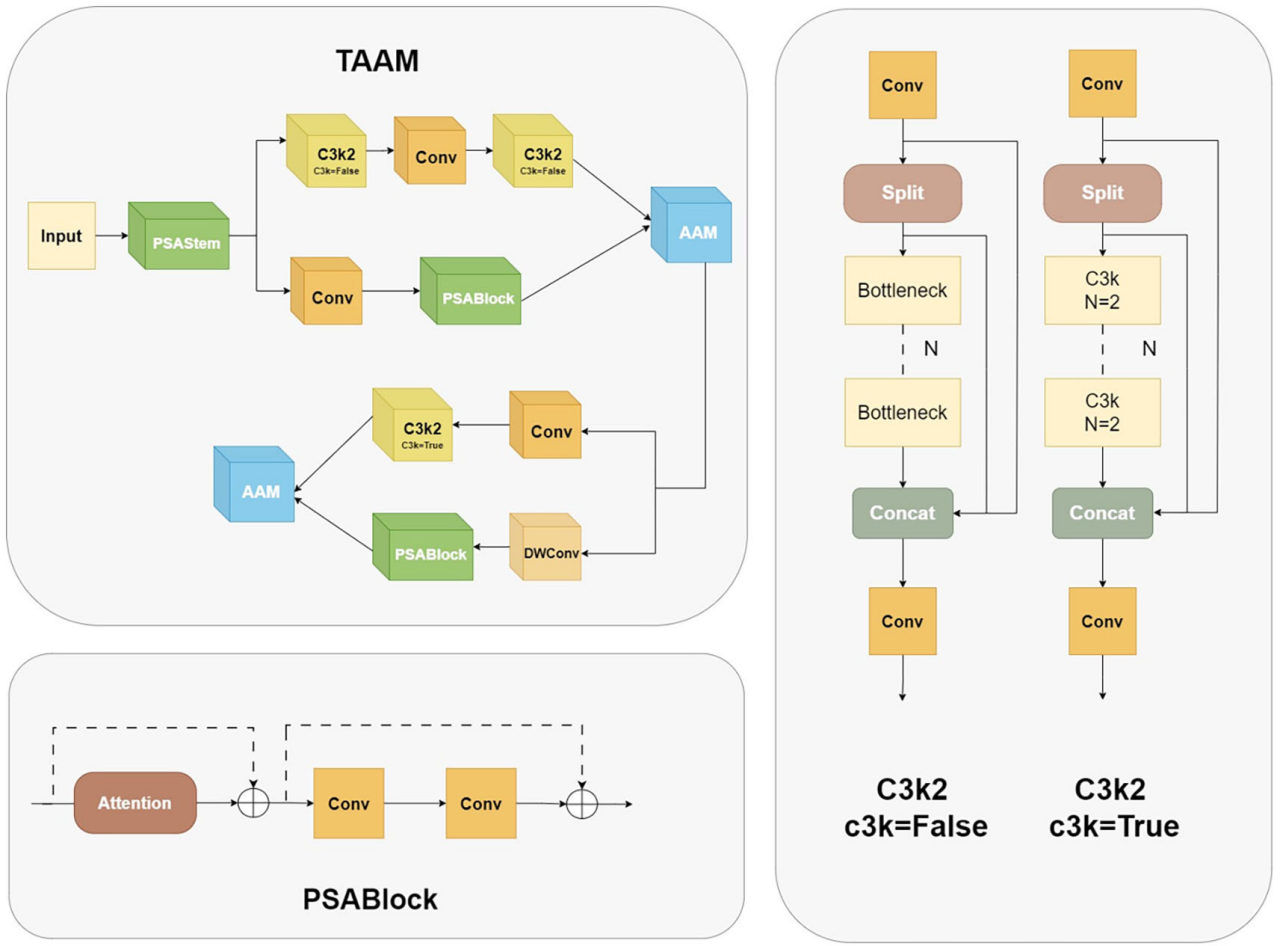
TAAM internal structure diagram.

The present study focuses on the problems of insufficient feature extraction of small-scale targets and redundant shallow computations in the YOLOv11 backbone for leaf lesion detection. It proposes a novel dual-branch adaptive alignment module, the Two-branch Adaptive Alignment Module (TAAM), as shown in **Figure 4**. The module under discussion achieves efficient computation through a multi-level feature sharing architecture. Firstly, the Pointwise Spatial Attention Stem (PSAStem) is utilised as the shared initial extraction layer, which pre-calibrates the input features through 1×1 pointwise convolution and an adaptive mechanism, thereby enabling the network to form dynamic spatial focusing capabilities at the input stage. Subsequently, the feature maps are processed through dual paths. The primary pathway integrates two C3k2 modules,each containing three standard 3×3 convolutions with 64 output channels, and a standard 3×3 convolution to preserve intricate features. The secondary pathway employs a 1×1 dimension-reducing convolution (reducing channels by a factor of 2) and subsequently connects to the optimised PSABlock. The initial feature extraction module combines pointwise convolution and spatial attention mechanisms, enabling the network to prioritise key regions in the input image and enhance the dynamic focusing ability on key spatial regions in the input stage while maintaining computational efficiency.

**Figure 5:**
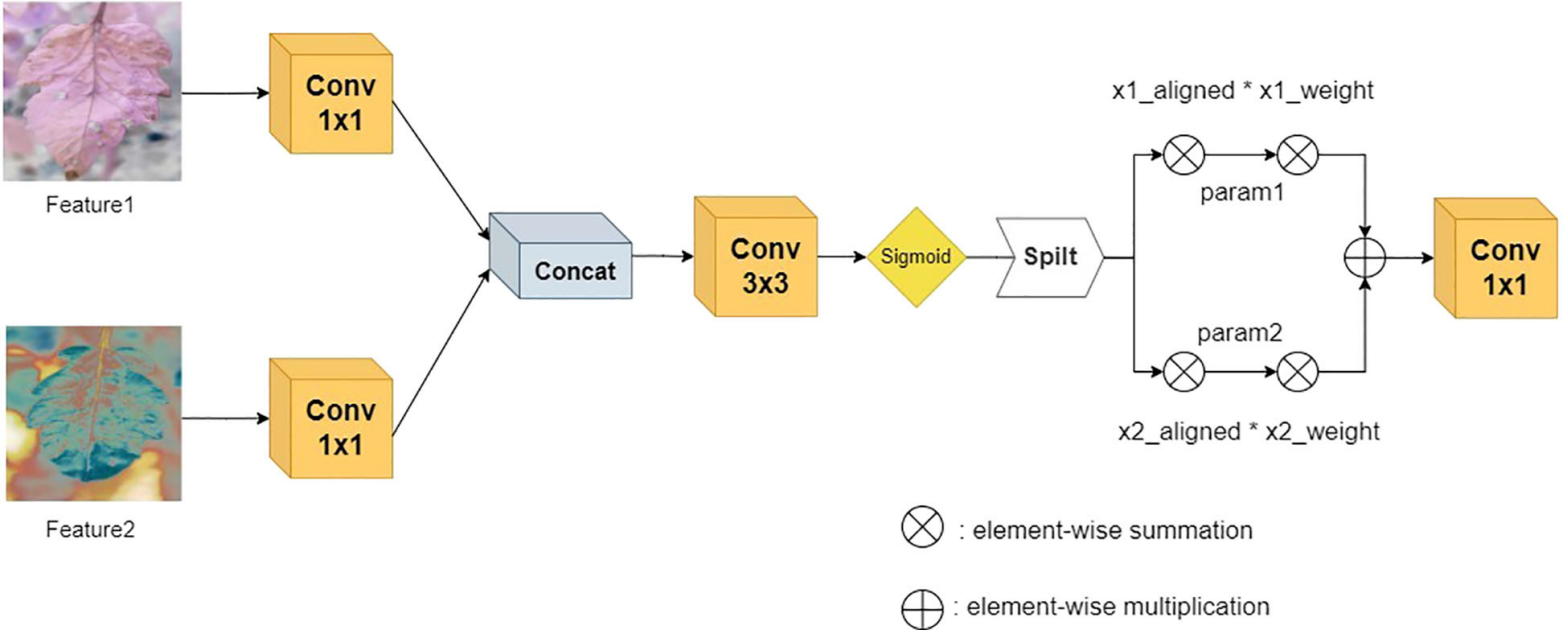
AAM structure diagram.


After the dual-branch channels process the features in parallel, they are connected to the Adaptive Alignment Module (AAM), as shown in **Figure 5**. The input dual-channel features F_1_ and F_2_ are first compressed in the channel dimension through 1×1 convolution layers to obtain F_1_’ and F_2_’, and then adaptive alignment weights - Adaptive Align Weight are generated through the cross-branch feature interaction layer to dynamically balance the contribution of the two paths. They are input into a 3×3 dilated convolution to capture long-range context dependencies and generate dynamic path selection weights α through the Sigmoid activation function. After spatial alignment of the two paths, the key region responses are enhanced through element-wise multiplication, as shown in Equations 2 and 3, where σ represents the Sigmoid activation function and W_α is a learnable weight matrix. Finally, multi-scale feature complementarity is achieved through element-wise addition, and the results are merged and output.

(2) $$\begin{array}{c} x1{\_}_{weight}=\sigma (W_{\alpha }^{1}⊗F_{1}^{'})\;\;\;\;\alpha ,\sigma \in [0,1] \end{array}$$

(3) $$\begin{array}{c} x2{\_}_{weight}=\sigma (W_{\alpha }^{2}⊗F_{2}^{'})\;\;\;\;\alpha ,\sigma \in [0,1] \end{array}$$

The innovation of this module can be attributed to the following: The following two mechanisms are introduced: 1) A dynamic weight adjustment mechanism is employed to optimise branch weights in real time based on the semantic distribution of the input features. This mechanism alleviates the spatial offset problem of heterogeneous features, such as misalignment interference between leaf lesions and healthy tissues. 2) Through the collaborative design of dilated convolution and gated attention, computational redundancy is reduced while local details and global pathological patterns are jointly modelled.

#### 2.2.3 Faster-gated linear unit

**Figure 6:**
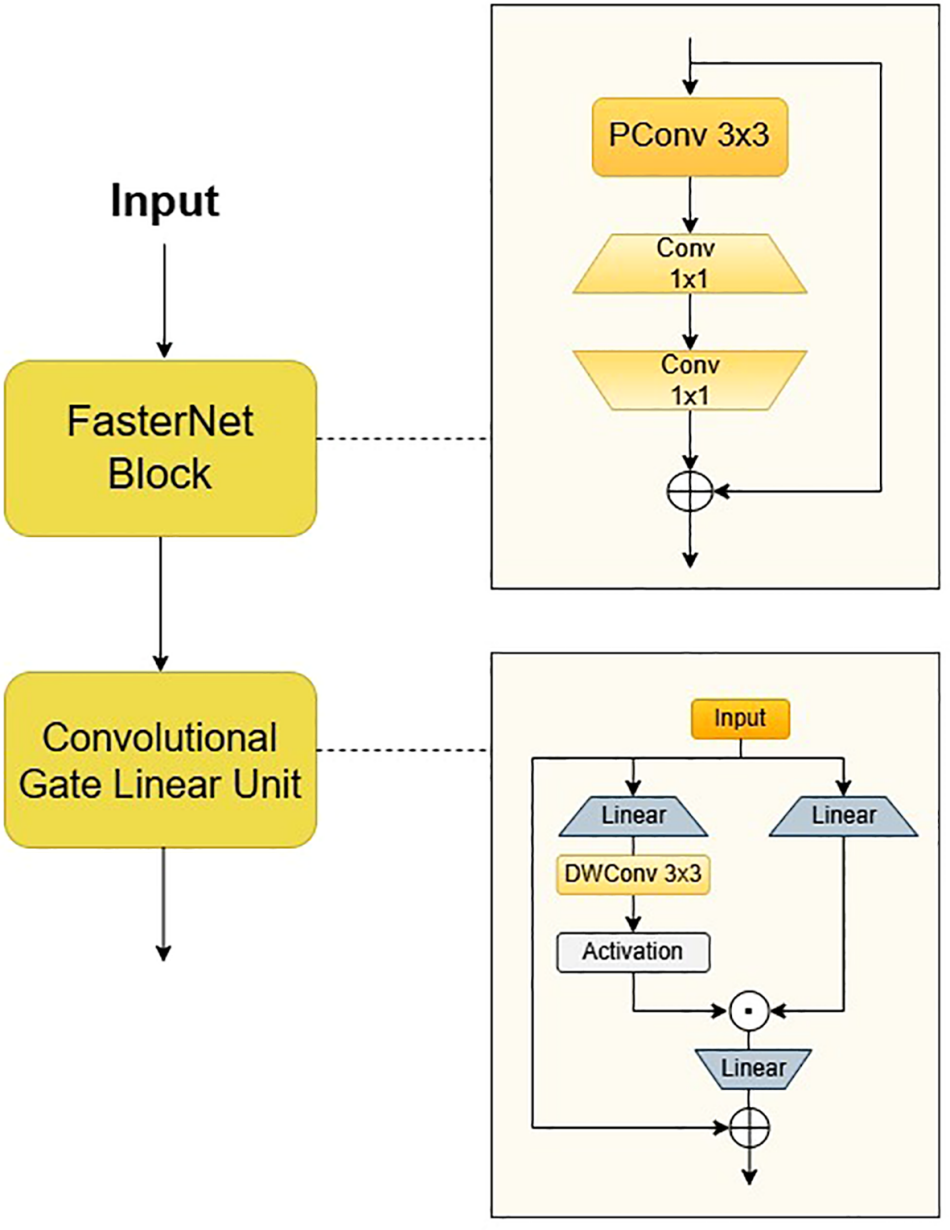
Faster GLUDet module structure diagram.

The neck network of YOLOv11 employs depthwise separable convolution and channel pruning strategies, which have been shown to enhance the recall rate of small targets while concurrently reducing the model’s parameters. Tomato leaf diseases frequently manifest as minute spots, and the receptive field of the P5 layer in the feature pyramid is overly extensive, which may impede the learning of small target features. Secondly, when the brown necrotic spots of tomato late blight are similar in colour to the soil, the feature pyramid network may confuse the target with the background. In order to address these issues, we propose Faster-GLUDet, whose core lies in enhancing the model’s ability to extract disease features in complex backgrounds through a gating mechanism while maintaining model lightweight. The Faster-GLUDet module integrates FasterNetBlock and Convolutional GLU (Chen et al., 2023; Shi, 2023), as illustrated in **Figure 6**.

The module’s primary function is the extraction of feature units through the utilisation of FasterNet Block, employing a 3x3 Partial Convolution to extract spatial features from a quarter of the input channels. This approach results in a 25% reduction in computational load when compared with traditional convolution, while retaining edge detail information. Subsequently, two 1x1 convolutions are connected to perform channel dimension reduction and feature fusion, thereby ensuring the effective preservation of multi-scale disease features. In order to enhance the feature interaction between modules, a dynamic gated feature enhancement unit, known as the Convolutional Gated Linear Unit (ConvGLU), is connected after the Faster Block. The model employs a dual-branch convolution to generate feature maps and gating signals, dynamically suppressing background noise and enhancing the response in the lesion area through element-wise multiplication. In the gating branch of the traditional gated linear unit (GLU), ConvGLU decomposes the standard 3x3 convolution into a cascade structure of depthwise convolution (DWConv) and pointwise convolution (PWConv), and combines a linear projection layer and GELU activation function to construct a lightweight feature enhancement path. A lightweight 3x3 depthwise convolution operation is introduced prior to the activation function in order to construct a gating channel attention mechanism based on neighbourhood features. The design converts global channel attention into local context-aware dynamic weight adjustment through the local receptive field characteristics of the convolution kernel. This retains the important information filtering ability in the channel dimension while significantly reducing computational complexity. ConvGLU employs convolution operations to capture local features in the image, thereby enhancing its efficacy in processing local information in comparison to traditional FFN. It is also capable of adaptively enhancing small target features.

The integration of Faster-GLUDet has been demonstrated to enhance the model’s feature extraction and expression capabilities to a considerable extent. The lightweight design of FasterBlock provides low-latency input for CGLU, while the gated weight generation module of CGLU further optimises multi-scale feature interaction, enabling the model to maintain lightweight while enhancing the diversity and hierarchy of features. This, in turn, helps better capture the details and context information of the target object. The system has been engineered to achieve dynamic regulation of feature channels, thereby further enhancing the semantic segmentation and spatial understanding capabilities of features. The combination of these two approaches has been shown to more effectively fuse multi-scale features and alleviate the problem of information loss, significantly improving the model’s performance in challenging plant disease recognition tasks.

#### 2.2.4 Multi-scale decoupling head

**Figure 7:**
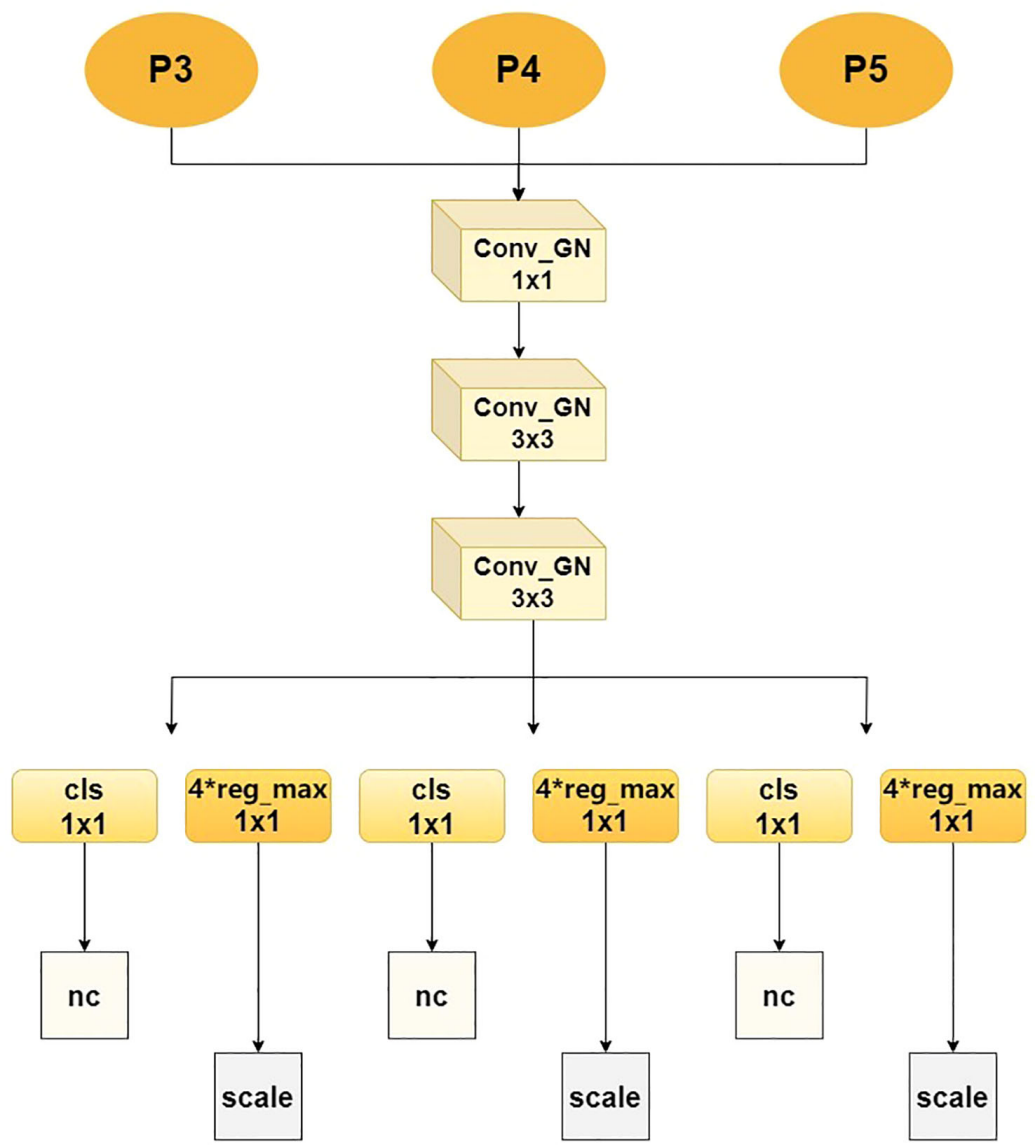
MDH detection head structure.

In order to address the issue of uneven detection accuracy of traditional detection heads for small and large-scale lesions, the Multi-scale Decoupling Head (MDH) has been proposed, as illustrated in **Figure 7**. The core process is as follows: MDH receives three different-scale feature maps - P3, P4 and P5 - from the Feature Pyramid Network (FPN) in parallel, which respectively carry high-resolution details, medium-scale information and large receptive field context, thereby constructing a multi-scale perception foundation. The features of each scale first enter a unified feature enhancement pathway, which is composed of a series of grouped normalized convolutional modules: First, the channel dimension is adjusted and fused through a 1×1 Conv_GN, and then two 3×3 Conv_GN modules are continuously used to enhance the spatial feature expression. At the same time, group normalization is utilized to ensure the stability of the model under small-batch training. After feature enhancement, the network flow is completely decoupled into two independent branches dedicated to their respective functions: The classification branch precisely extracts features through two consecutive 1×1 convolutional layers and ultimately outputs a probability graph with a dimension of nc, accurately determining the category of the target within each anchor box; The regression branch adopts the same structure, but its output dimension is 4 × reg_max, which indicates that it uses an advanced distributed focus loss mechanism. By predicting the discrete distribution of bounding box coordinates, it greatly improves the accuracy of lesion location, where reg_max defines the flexible maximum value of the distribution. Ultimately, the outputs of the two branches are respectively normalized and integrated through a scale layer to generate the final detection results.

## 3 Analysis of experimental results

### 3.1 Experimental platform and parameter settings

In this study, the image input size is set to 640×640 pixels. To accelerate the convergence speed, the initial learning rate is set to 0.01, the stochastic gradient descent algorithm (SGD) is used for training, the weight decay coefficient is set to 0.0005, the momentum factor is set to 0.937, a total of 200 periods, and the size of the training batch is set to 32 times, and the workers are set to 12. All the experiments are performed on a Linux server. All the experiments are realized on a Linux server, and the specific configuration of the experimental environment is shown in **Table 2**.

**Table 2:** Experimental environment configuration.

Environment Configuration	Parameters
GPU	2*A100(80GB)
CPU	Intel(R)Xeon(R)Gold 6148 CPU @2.40GHz
Development environment	PyCharm 2023.2.5
Language	Python 3.8.10
Framework	PyTorch 2.0.1
Operating platform	CUDA 11.8
Operating System	Linux

### 3.2 Analysis and evaluation of the identification results

#### 3.2.1 Evaluation index

In this paper, the metrics Precision, Recall, and mAP are utilised to evaluate the detection performance of the model. TP, FP, and FN represent the number of true positive, false positive, and false negative samples, respectively. C denotes the set of object categories, and |C| is the total number of categories. As shown in Equations 4, 5 and 6


(4) $$\begin{array}{c} Precision=\frac{TP}{TP+FP} \end{array}$$

(5) $$\begin{array}{c} Recall=\frac{TP}{TP+FN} \end{array}$$

(6) $$\begin{array}{c} mAP=\frac{1}{\left|C\right|}\sum_{c\in C}AP(c) \end{array}$$

P denotes the proportion of correctly detected disease samples among all positive detections, thereby reflecting the model’s capacity to avoid false positives. R signifies the proportion of correctly detected disease samples among the actual existing disease samples, thus measuring the model’s ability to reduce false negatives. AP quantifies the detection performance of the model for a single disease category by calculating the area under the precision-recall curve. mAP is the average of APs for all categories, and a higher mAP indicates that the model’s detection effect on various disease categories is more balanced and accurate.

#### 3.2.2 Comparative experiments of different models

**Figure 8:**
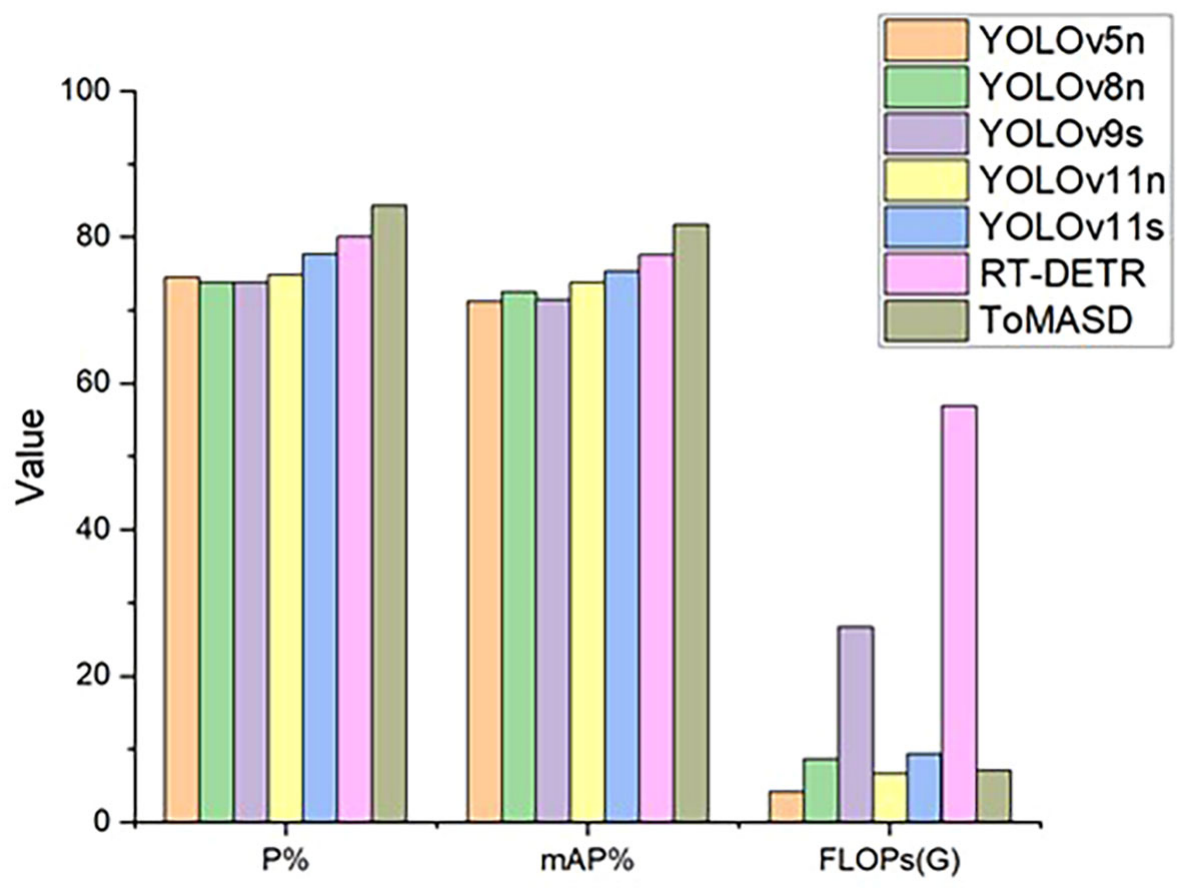
Performance bar charts of six models.

**Figure 9:**
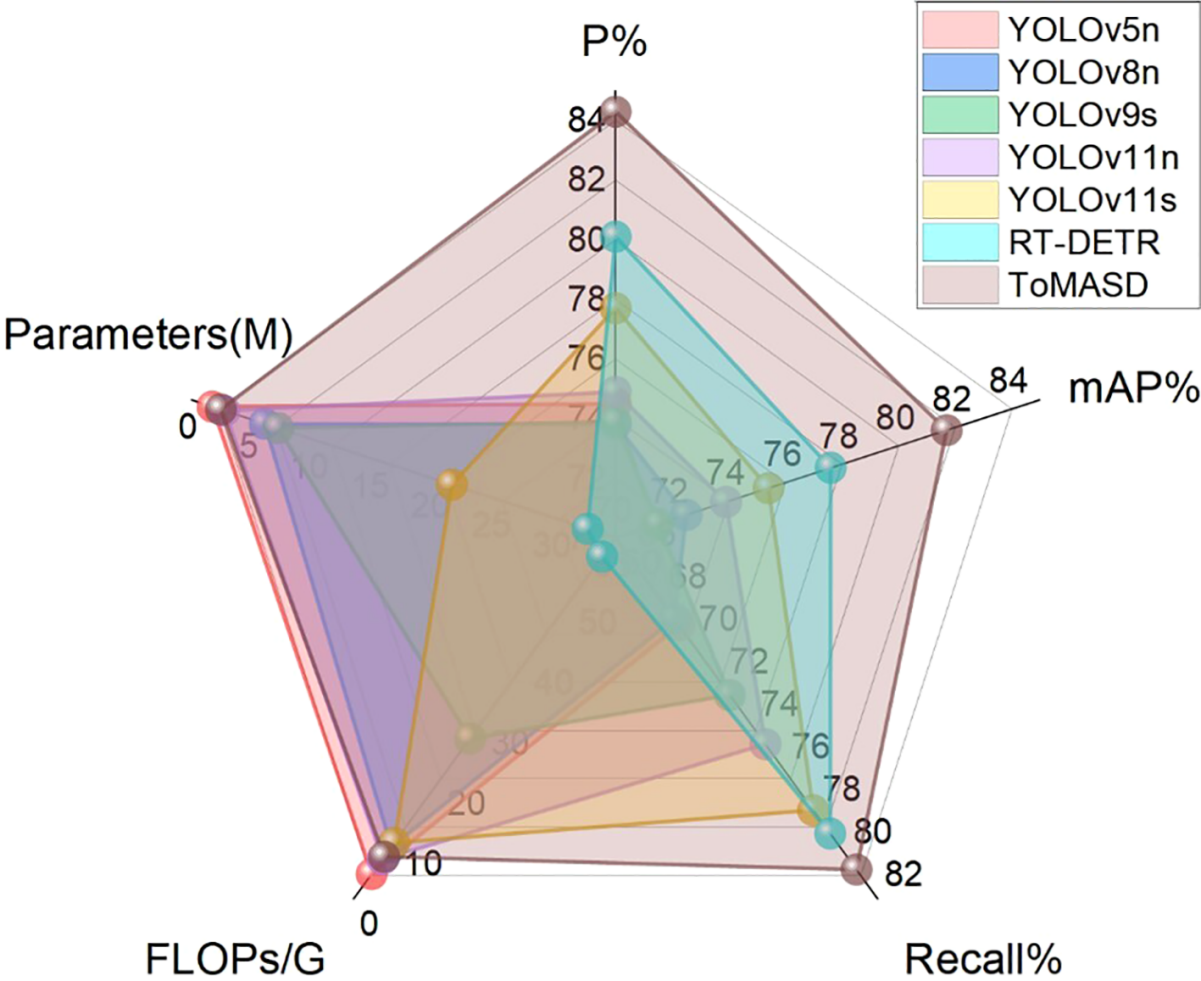
Comprehensive comparison of performance of six models.

To comprehensively evaluate the detection and generalization performance of the proposed ToMASD model, we conducted extensive comparative experiments with thirteen state-of-the-art object detection models on the same tomato leaf disease dataset. As summarized in **Table 3**, ToMASD achieved the highest scores in both precision and mAP, significantly outperforming all other contenders. RT-DETR achieves high accuracy but at the cost of high computational complexity, making it unsuitable for edge deployment. In comparison with the unimproved YOLOv11n, ToMASD has increased P, mAP, and Recall by 6.6%, 7.8%, and 5.9%, respectively, demonstrating its superior ability in target localization and classification in complex scenarios. Despite the fact that YOLOv11n exhibits a modestly diminished number of FLOPs in comparison with ToMASD, a notable deterioration in accuracy is evident, suggesting the potential for optimisation shortcomings within the feature ex-traction process. Despite the advantages in parameter quantity and computational cost of YOLOv5n and YOLOv11n, their accuracy still lags significantly behind ToMASD, further confirming the dual improvements in accuracy and recognition performance of the ToMASD model. Whilst sustaining its position of being lightweight, it has considerably surpassed the constraints of prevailing algorithms in the trade-off between ac-curacy and computational resource consumption. As illustrated in **Figure 8**, a performance comparison of the six models with the highest mAP is presented. A selection of six algorithms with comparable performance was made for the purpose of a comprehensive comparison, as illustrated in **Figure 9**. The performance of the metric is optimised by the distance of each axis of the curve from the intersection point. The area enclosed by the curve is positively correlated with the strength of the algorithm’s comprehensive performance. The comparison results demonstrate that the ToMASD model proposed in this paper exhibits advantages in all metrics, not only improving performance but also achieving lightweight, thus rendering it more suitable for practical scenarios.

**Table 3:** Comparison of object detection results of different algorithms.

Models	P%	mAP%	Recall%	FLOPs/G	Parameters
SSD (Liu et al., 2016)	76.5	72.3	70.7	200.6	4.48×10^7^
YOLOv3-tiny (Redmon and Farhadi, 2018)	73.6	66.8	61.1	18.9	1.21×10^7^
YOLOv5n	74.5	71.3	69.1	4.2	1.76×10^6^
YOLOv6 (Li et al., 2022)	69.1	68.2	65.6	11.1	4.23×10^6^
YOLOv7-tiny (Wang et al., 2022)	70.0	69.4	68.9	13.2	6.07×10^6^
YOLOv8n	73.8	72.5	68.8	8.7	3.00×10^6^
YOLOv8s	77.8	75.6	77.5	28.6	1.12×10^7^
YOLOv9t (Wang et al., 2024b)	69.1	67.8	70.1	7.9	2.01×10^6^
YOLOv9s	73.9	71.5	72.4	26.7	7.17×10^6^
YOLOv10n (Wang et al., 2024a)	76.2	70.8	67.9	8.2	2.69×10^6^
YOLOv11n	74.9	73.9	74.7	6.7	2.76×10^6^
YOLOv11s	77.7	75.4	77.8	9.4	2.15×10^7^
RT-DETR	80.1	77.6	78.9	56.9	3.27×10^7^
ToMASD	84.3	81.7	80.6	7.1	2.46×10^6^

To verify the performance of the TAAM module, this study systematically evaluated the effects of four mainstream attention modules as the Stem, as shown in **Table 4**. PSA achieved the best balance between accuracy and computational efficiency, reducing the computational complexity by 17.7% compared to the ECA module with the sec-ond-highest accuracy, verifying the performance of PSA.

**Table 4:** Comparison of different attention modules.

PSA	CBAM	ECA	ELAN	P%	FLOPs/G
✓				77.4	20.9
	✓			72.8	20.9
		✓		76.2	25.4
			✓	73.6	23.1

**Figure 10:**
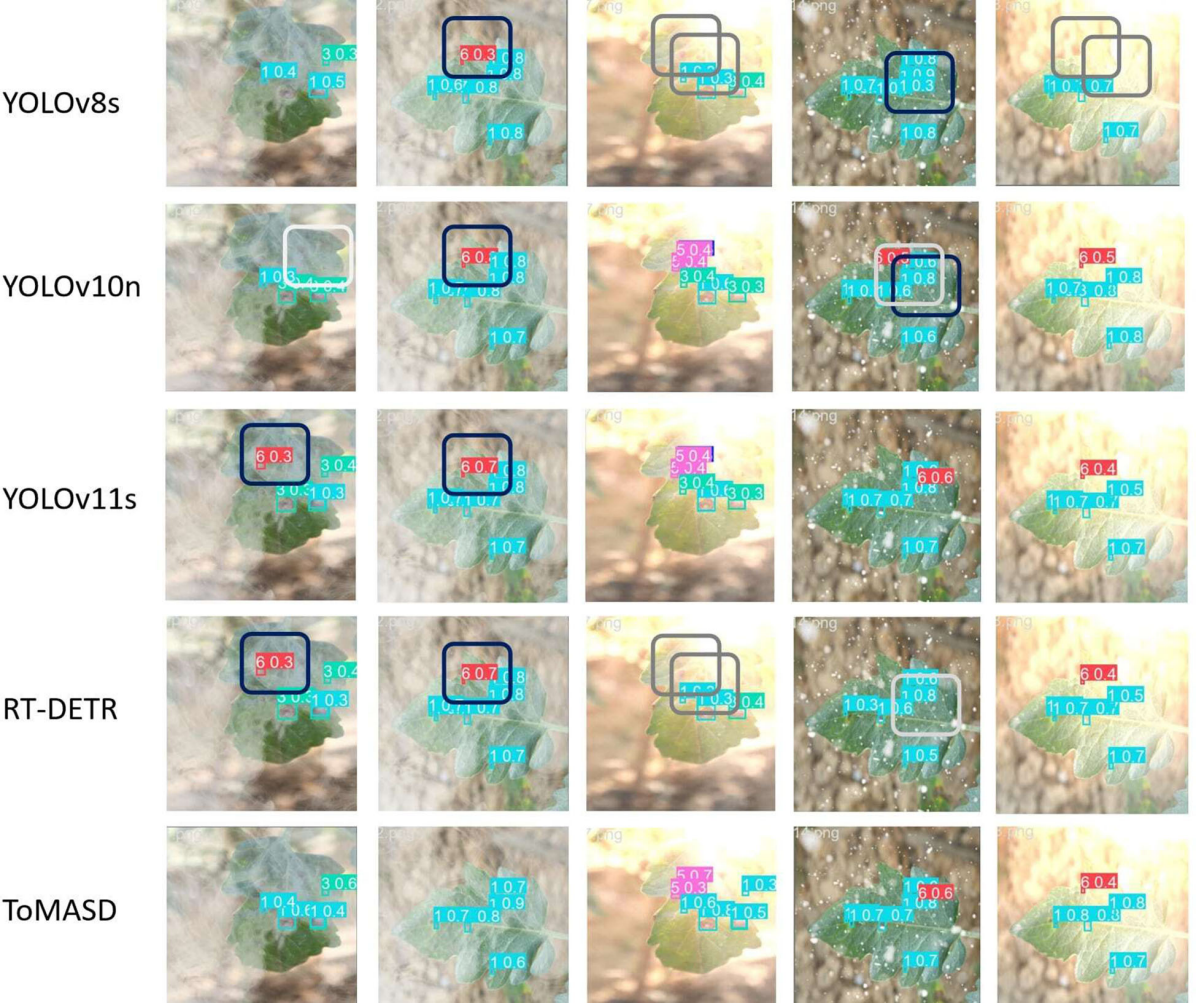
Detection results of five models under different weather conditions (gray boxes indicate missed detections and black boxes indicate false positives).

**Figure 11:**
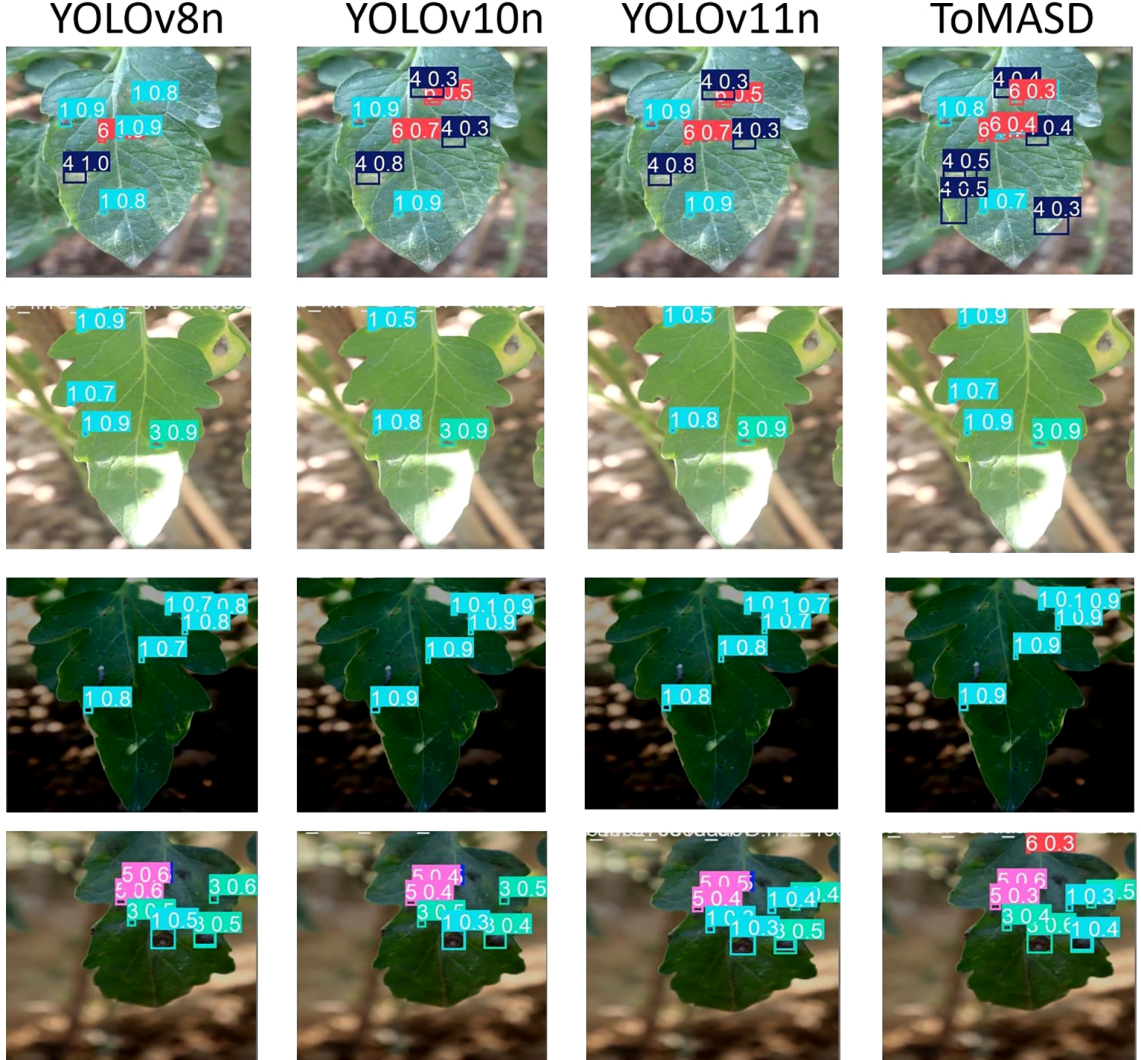
Detection results of the four models in a conventional detection environment.

**Figure 12:**
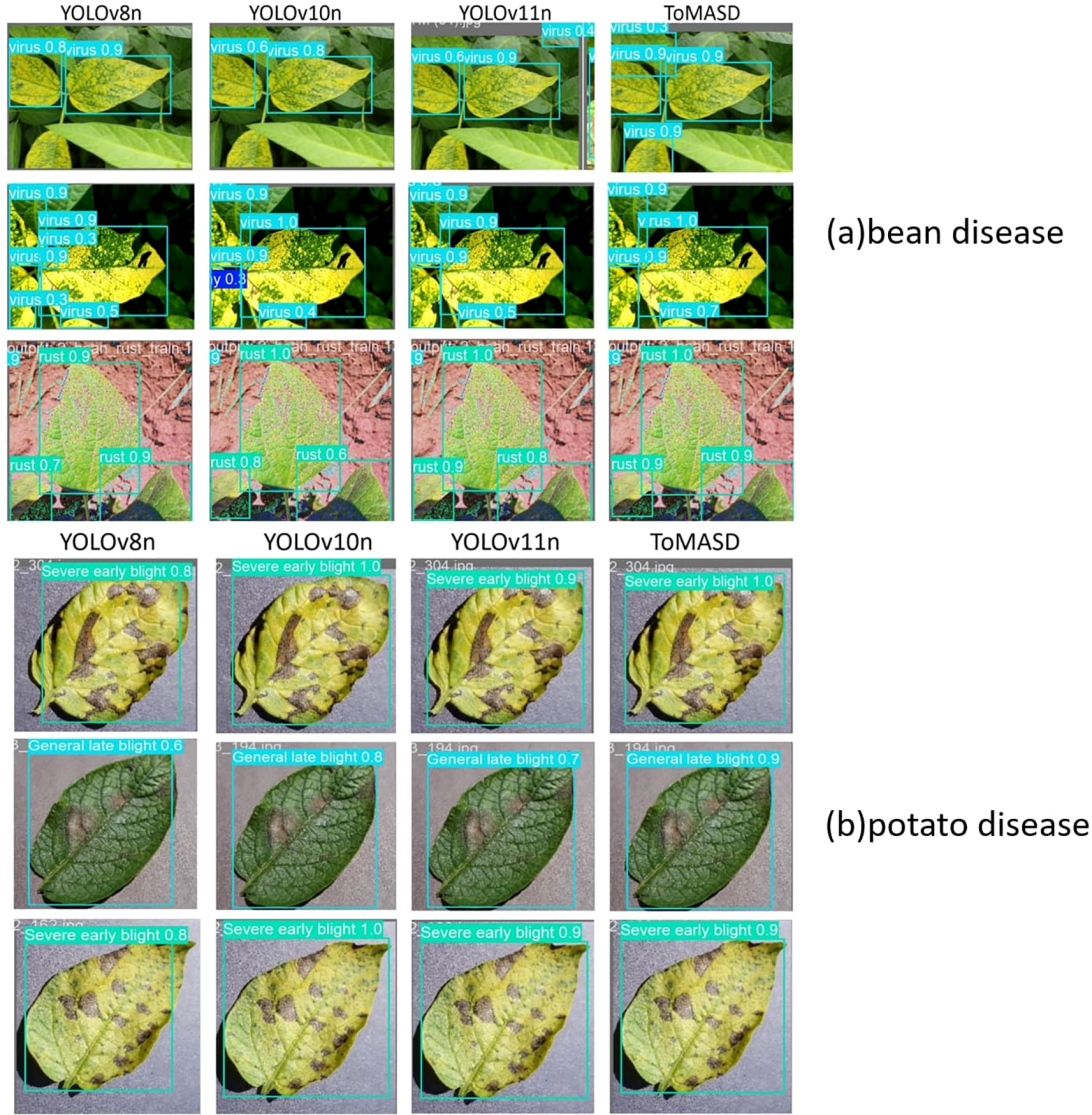
The detection results of leaf diseases of common beans and potatoes by ToMASD: **(a)** bean disease, **(b)** tomato disease. The models from left to right are YOLOv8n, YOLOv10n, YOLOv11n.

**Figure 13:**
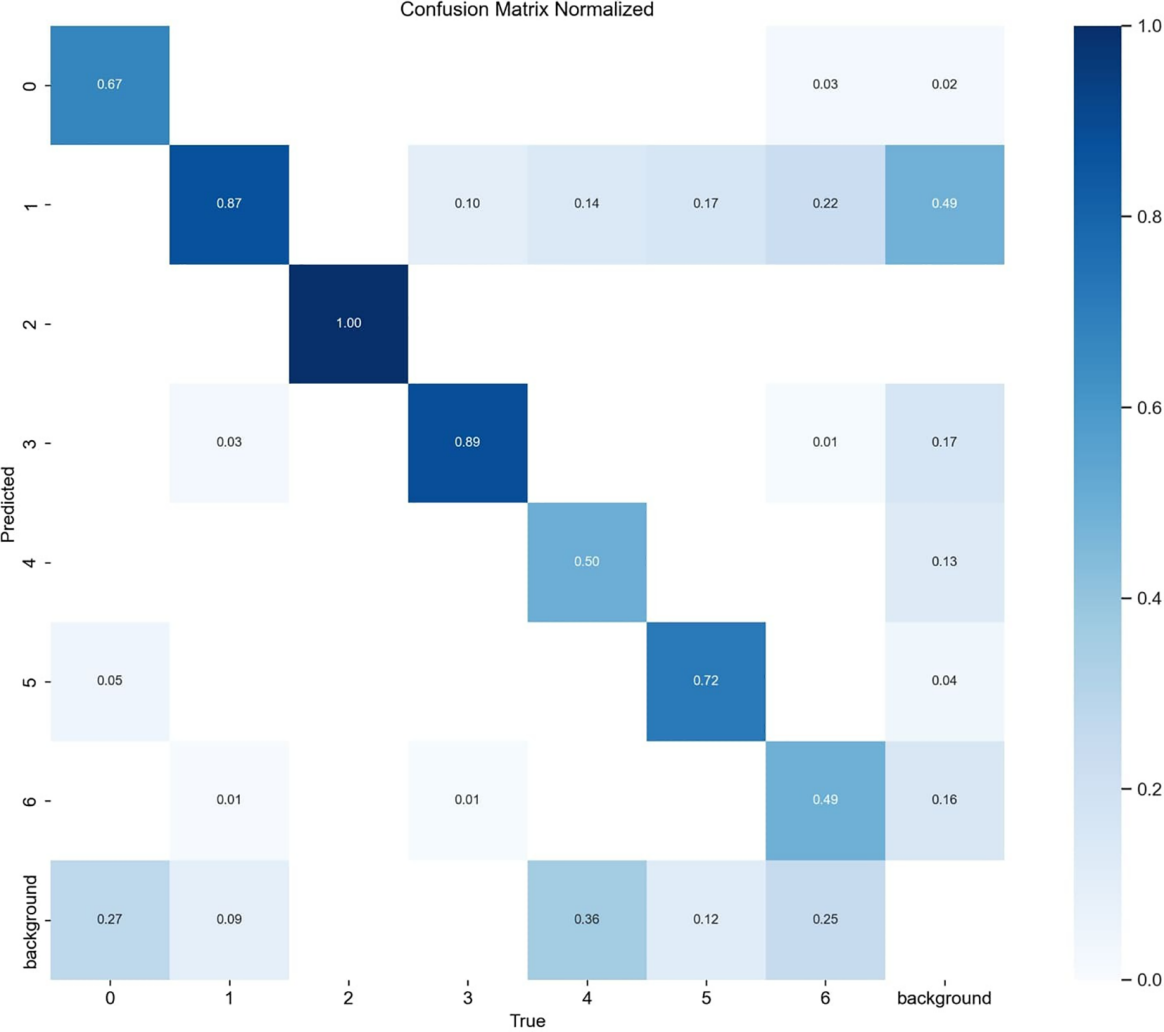
Confusion matrix.

**Figure 10** shows the detection performance of the five models with the highest accuracy under different weather conditions, where gray boxes indicate missed detec-tions and black boxes indicate false positives. Under foggy conditions, except for To-MASD, the other four models misjudged fog points as diseases. When the light intensity was too high, the comparison models also failed to effectively suppress the exposed areas, resulting in missed detections of some real lesions. **Figure 11** shows the detection results of the four lightweight models in conventional environments. Comprehensive comparative analysis shows that ToMASD exhibits the most superior performance under various complex weather conditions and has superior feature extraction and denoising capabilities in real agricultural environments. **Figure 12** shows ToMASD’s detection of potato and common bean leaf diseases, and the experimental results show that ToMASD maintains high accuracy in the cross-crop task, achieving 92.1% and 93.5% accuracy in the detection of bean and potato leaf diseases, respectively, demonstrating its efficient generalization ability and transferability nature of cross-species training. **Table 5** shows the recognition accuracy of ToMASD for different spots, **Figure 13** shows the confusion matrix of this experiment, the model still maintains stable recognition performance in the category imbalance dataset, the recognition accuracy of Late Blight, Early Blight is close to 90%, Leaf Mold has a similar chromaticity of the yellow spot and the healthy tissues, which leads to the relatively low detection accuracy. Bacterial Spot and Target Spot have similar water-damaged spot characteristics, but the model still achieved 82.1% and 74.1% mAP values through multi-scale texture analysis, indicating the effectiveness of the feature decoupling mechanism.

**Table 5:** Evaluation indicators for different diseases.

Evaluation index	Bacterial Spot	Early Blight	Healthy	Late Blight	Leaf Mold	Target Spot	Black Spot
P%	85.4	90.1	99.8	85.2	71.9	77.8	80.3
mAP%	82.1	88.0	98.7	82.4	69.5	74.1	76.1

#### 3.2.3 Ablation experiment

The proposed ToMASD model is based on YOLOv11n and has been optimised by introducing TAAM, Faster-GLUDet, and MDH. In order to evaluate the performance of each optimisation module, an experiment was conducted using the variable control method. The training and testing were carried out on the same dataset and training parameters, and the results are shown in **Table 6**. It is evident that Model C attained an mAP% of 77.9%, while concurrently sustaining a minimal computational cost. However, Model D, which introduced TAAM and Faster-GLUDet, exhibited an increase in computational cost to 12 FLOPs/G, attributable to parameter redundancy, yielding an accuracy of only 79.2%. The experiments indicated that the joint application of TAAM and MDH caused feature decoupling conflicts. The Model E achieved an 82.1% P% and 80.1% mAP%, thereby demonstrating the viability of multi-module collaborative optimisation through its lightweight design. The ToMASD model proposed in this paper was found to achieve the optimal balance in parameters, computational efficiency, and performance. In comparison with the baseline model A, it enhanced the accuracy by 9.4% whilst escalating the computational cost by a mere 0.4G. This outcome serves to demonstrate the efficacy of the collaborative design of multi-dimensional attention mechanisms and lightweight architectures for object detection tasks.

**Table 6:** Results of model improvement ablation experiment.

Model	TAAM	Faster-GLUDet	MDH	P%	mAP%	Parameters	FLOPs/G
A	✓			77.4	72.6	1.75×10^7^	20.9
B		✓		79.9	74.4	2.45×10^6^	6.1
C			✓	80.3	77.9	1.84×10^6^	4.3
D	✓	✓		79.2	72.3	4.46×10^6^	12.0
E		✓	✓	82.1	80.1	2.36×10^6^	6.5
F	✓		✓	78.8	77.1	2.69×10^6^	7.6
ToMASD	✓	✓	✓	84.3	81.7	2.46×10^6^	7.1

#### 3.2.4 Heat map visualization analysis

**Figure 14:**
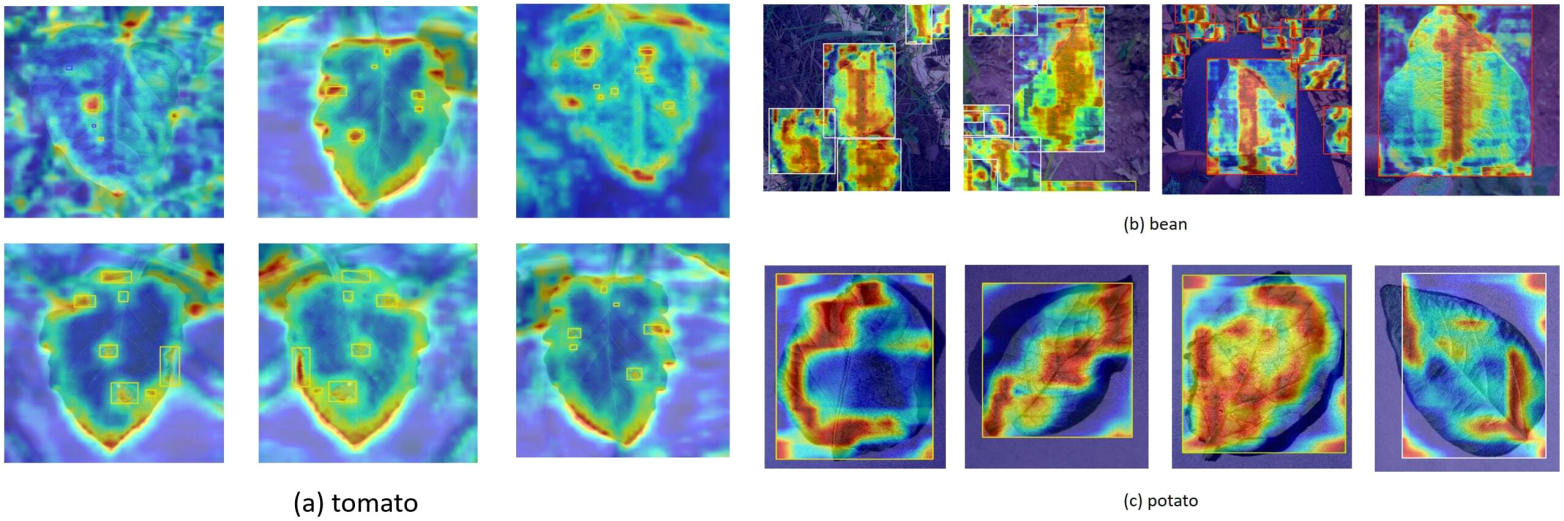
Feature visualization of leaf disease patterns across different crops: **(a)** tomato; **(b)** common bean; **(c)** potato.

The present study employed the gradient-weighted class activation mapping technique (Selvaraju et al., 2017) to visualise the small target detection mechanism of the ToMASD model (**Figure 14**). The experimental findings demonstrate that the tomato leaf disease detection model, based on transfer learning, exhibits adapted feature capabilities in different crop disease recognition tasks. When the model is transferred from the source domain of tomatoes to the target domain of common beans, the heatmap analysis indicates that the lesion areas can still be effectively captured, although the the extent of the activated regions of highlighted areas is lower than that in the source domain. This suggests that the model has initially acquired the ability to locate disease spots across species through transfer learning. When the heatmap is transferred to potatoes with more distinct morphological features, more concentrated highlighted areas are shown, which may be related to the reticulate vein structure of potato leaves, thereby enhancing the distinguishability of texture features. It is noteworthy that the heatmaps of all three crops demonstrate a substantial contrast between the lesion areas and healthy tissues, thereby confirming that the model, while retaining key pathological features, has achieved adaptive adjustments to different crop leaf diseases through weight transfer. This cross-species disease recognition capability provides a feasible solution for intelligent diagnosis of multiple crop pests and diseases under resource-limited conditions.

## 4 Discussion

### 4.1 Resource identification initiative

The present study proposes a detection model, ToMASD, which integrates multi-scale feature fusion and dynamic attention mechanisms, with a view to addressing the challenges of tomato leaf disease detection in complex agricultural environments. The prevailing field of agricultural disease detection frequently grapples with challenges such as leaf occlusion, a wide spectrum of lesion morphologies, and intricate lighting conditions. Conventional methodologies are predicated on manually designed features, which are challenging to adapt to the multi-scale representation requirements of dynamic field environments. Despite its promising performance, ToMASD has several limitations: real-time deployment, Although lightweight, the model may still struggle on low-end edgedevices. Knowledge distillation or quantization could further compress the model. The research under discussion addresses the issue of misalignment between diseased and healthy tissues by designing a Two-branch Adaptive Alignment Module with a dynamic weight allocation mechanism. Secondly, the Faster-GLUDet module enhances noise suppression capabilities while maintaining a lightweight model through a local context-aware gating unit. The innovative architecture of the model integrates deep convolution and pointwise convolution to construct gating signals, thereby achieving a substantial reduction in background false detection rates, particularly in conditions characterized by foggy and strong light. It is evident that the multi-scale decoupled detection head (MDH) successfully achieves balanced detection of both small and diffuse lesions. This is achieved through the implementation of group normalisation and the establishment of independent classification and regression branches. A series of ablation experiments were conducted, the results of which demonstrate that MDH enhances the mAP value of imbalanced datasets by 7.8%, particularly with regard to the recognition accuracy of low-contrast diseases such as leaf mold.

Despite the fact that ToMASD demonstrates superiority over existing models in a number of metrics, there is still scope for enhancement. For instance, the mAP for Black Spot detection is only 76.1%, indicating that the ability to distinguish similar texture diseases needs further optimization. It is recommended that future research explore the potential of knowledge distillation techniques to further compress the model size. In addition, the combination of spatio-temporal features could enhance the prediction of disease spread dynamics. In conclusion, ToMASD has been demonstrated to provide an efficient and reliable solution for precise disease diagnosis in complex agricultural scenarios through the collaborative design of multi-dimensional attention mechanisms and lightweight architectures. This suggests that there is significant practical value and potential for promotion.

This study further validates the potential of ToMASD model for transfer learning in cross-crop disease detection. The weights of the tomato disease detection model were migrated to the potato and common bean tasks, and the cross-crop features were fused by domain adaptive layer state, and an adversarial feature decoupling strategy was used to suppress the inter-domain distribution bias. Experiments show that the migration model achieves 93.5% and 92.1% accuracy values on the potato and common bean test sets, respectively. The technical innovation is that by decoupling the cross-crop shared features and crop-specific features, the model overcomes the problem of confusing the small brown spots of bean rust with the background leaf vein texture while retaining the accuracy of tomato disease detection, supporting the joint monitoring of multi-crop diseases. The results show that the lightweight architecture and domain adaptive mechanism of ToMASD provide a cross-species generalization paradigm for building a general-purpose agricultural disease monitoring system, which significantly reduces the model development cost for multi-crop disease detection.

## 5 Conclusions

This study proposed ToMASD, a novel lightweight detection model, to address the critical challenges of tomato disease detection in complex agricultural environments. The core contributions of this work are threefold, each validated by extensive experimental results:

Firstly, to mitigate the feature attenuation of small lesions and the misalignment interference between diseased and healthy tissues, we designed the Two-branch Adaptive Alignment Module (TAAM). This module dynamically aligns cross-scale features, which was a key factor in achieving the overall mAP of 81.7%, a significant improvement over all baseline models.

Secondly, to enhance feature representation while suppressing complex background noise, we integrated the Faster-GLUDet feature enhancement unit. Its local context-aware gating mechanism effectively reduced false positives, as evidenced by the low misdetection rates of 6.3% in foggy and 9.8% in strong light conditions, while maintaining a low computational cost of only 7.1 GFLOPs.

Thirdly, to balance the detection accuracy between tiny spots and large lesions, we developed the Multi-scale Decoupling Head (MDH). By employing Group Normalization and independent task-specific branches, the MDH ensured balanced detection, which is reflected in the stable performance across all six disease categories, even under severe class imbalance.

Future research can be extended in three aspects: first, integrating hyperspectral imaging technology to enhance the characterisation of chromaticity gradient diseases; second, verifying the generalization of the model in economic crops such as chili peppers, grapes. based on the current migration learning framework; third, exploring the deployment scheme of edge computing to build a low-power field monitoring network in combination with LoRaWAN wireless transmission, to realize spatio-temporal prediction of disease spreading and precise prevention and control. The study is based on a lightweight architecture and a low-power edge computing deployment scheme. Through the deep integration of lightweight architecture and migration learning technology, this study provides a scalable solution for intelligent diagnosis of agricultural diseases, promotes the digital transformation of disease monitoring from single-crop scenarios to multi-species and multi-environment collaborative management, and assists the sustainable development of agriculture.

## Data Availability

Publicly available datasets were analyzed in this study. This data can be found here: https://gitcode.com/open-source-toolkit/829ec.
